# Medical and Nonmedical Information during Multidisciplinary Team Meetings in Cancer Care

**DOI:** 10.3390/curroncol28010098

**Published:** 2021-02-23

**Authors:** Jessica Wihl, Linn Rosell, Tobias Carlsson, Sara Kinhult, Gert Lindell, Mef Nilbert

**Affiliations:** 1 Department of Clinical Sciences Lund, Division of Oncology, Lund University, 22381 Lund, Sweden; jessica.wihl@skane.se (J.W.); linn.rosell@med.lu.se (L.R.); 2 Regional Cancer Centre South, Region Skåne, 22381 Lund, Sweden; tobias.carlsson@skane.se; 3 Department of Hemathology, Oncology and Radiation Physics, Skåne University Hospital, 22185 Lund, Sweden; sara.kinhult@skane.se; 4 Department of Surgery, Skåne University Hospital, Lund University, 22185 Lund, Sweden; gert.lindell@skane.se; 5 Clinical Research Centre, Hvidovre Hospital and Copenhagen University, 2650 Hvidovre, Denmark; 6 Danish Cancer Society Research Centre, 2100 Copenhagen, Denmark

**Keywords:** tumor board, cancer conference, decision-making, patient-centered, comorbidity, occupation

## Abstract

Background: Multidisciplinary team (MDT) meetings provide treatment recommendations based on available information and collective decision-making in teams with complementary professions, disciplines and skills. We aimed to map ancillary medical and nonmedical patient information during case presentations and case discussions in MDT meetings in cancer care. Methods: Through a nonparticipant, observational approach, we mapped verbal information on medical, nonmedical and patient-related characteristics and classified these based on content. Data were collected from 336 case discussions in three MDTs for neuro-oncology, sarcoma and hepato-biliary cancer. Results: Information on physical status was presented in 48.2% of the case discussions, psychological status in 8.9% and comorbidity in 48.5% of the cases. Nonmedical factors, such as family relations, occupation, country of origin and abode were referred to in 3.6–7.7% of the cases, and patient preferences were reported in 4.2%. Conclusions: Provision of information on comorbidities in half of the cases and on patient characteristics and treatment preferences in <10% of case discussions suggest a need to define data elements and develop reporting standards to support robust MDT decision-making.

## 1. Introduction

In cancer care, treatment recommendations based on collective multidisciplinary and multi-professional decision-making have developed into standard of care. Case discussions based on multidisciplinary team (MDT) meetings qualify treatment recommendations based on different skills and perspectives, provide best expert opinion in areas with limited evidence and support treatment recommendations adherent to evidence-based clinical guidelines. Though there is general support for case management through MDTs, data to support improved patient outcomes are scant and suggest that the effects may differ among various diagnostic areas [1,2]. To date, research on MDT-based decision-making has predominantly focused on meeting structure, organization, team contributions and factors that drive treatment recommendations [3,4]. 

Formulation of an individualized treatment recommendation is a complex process that requires a well-structured case presentation, access to relevant data, experts’ input to case discussions, integration of a multitude of information, skilled leadership, an open discussion climate and well-functioning teamwork. Consideration of patient characteristics has been suggested to lead to more robust MDT decisions with a higher likelihood of implementation of the treatment recommendations made [3,4,5]. Observations, however, suggest that case presentations and case discussions at MDTs tend to focus on clinical information and medical aspects with more limited consideration of patient characteristics [3,4,5,6,7,8,9,10]. Patient-related factors include medical information related to general health and performance status, comorbidity, risk factors, psychological factors, vulnerabilities, and nonmedical factors such as country of origin, supportive network, occupation, abode and patients’ preferences. 

We aimed to map provision and content of medical and nonmedical patient information in MDT meetings based on a nonparticipant, observational approach with correlation to patient and team characteristics. 

## 2. Study Design and Methods

### 2.1. Study Design and Setting

Prospective data collection was based on nonparticipants’ observations in three MDT teams that specialized in neuro-oncology, soft-tissue sarcoma and hepatobiliary cancer. The clinical setting was a Swedish University hospital with regional expert responsibilities in cancer care for a population of 1.9 million inhabitants. The teams had representatives from all relevant disciplines, including surgeon, oncologist, neurologist (in the neuro-oncology team), radiologist, pathologist and specialized nurse. All three teams held weekly meetings, and two teams included health professionals from regional hospitals who joined through video link. The teams had defined meeting structures and guidelines for the information required to refer patients to the meetings. The teams had, however, not applied formal frameworks for decision-making, used case presentation checklists or undergone formal evaluation or accreditation to assess MDT performance. 

### 2.2. Method

Between April and October 2019, we observed 30 MDT meetings with case discussions related to brain tumors, soft-tissue sarcomas and hepatobiliary cancers. The observations were conducted by three nonparticipant observers (two oncologists and one oncology nurse), all of whom had experience from observational MDT assessment in adjoining research projects. For each case discussed, data on sex, age and treatment recommendation (yes/no) were collected on a standardized study scheme. MDT meeting participants’ verbal statements were collected in the form of open notes for each case. Information and comments related to the cancer diagnosis, treatment and follow-up were disregarded, but all information on patient-related factors and characteristics such as symptoms, physical status, comorbidity, psychological status, occupation, abode, country of origin, patient preferences and other nonmedical information provided was noted. Information was collected during the case presentations as well as the case discussions. Comments and input from all team members were considered, but were not mapped in relation to the specialty or profession of team participants. Two authors (JW and MN) independently assigned the notes into the predefined categories with inspiration from a previous study in the field (Table 1) [11]. In discrepant cases the observers reread the texts to reach consensus. 

Double, independent registrations were performed by two observers at seven MDT meetings, which rendered duplicate data from 101/336 case discussions. Concordance was defined as both observers identifying a certain information and classifying it into a distinct category, though individual wording could differ somewhat between the observers’ notes. 

### 2.3. Statistics Analysis

Baseline characteristics are presented using descriptive statistics. We used four multiple logistic regression models to assess the relationship between available information related to comorbidity, physical status, psychological status and nonmedical information. The response variables equaled 1 if information was available and 0 if no information was available. Our independent variables of interest were the patients’ age, sex and MDT team. Adjusted odds ratios (OR) and corresponding 95% confidence intervals (CI) were used as inference for all variables in the models. CIs that did not include the null value were considered statistically significant. Data processing and statistical analysis were performed with the statistical software R [12]. 

### 2.4. Ethical Considerations

All data were handled anonymously and are presented at group level. The study was ethically reviewed and granted permission by the regional ethics committee at Lund University (registration numbers 2016-195 and 2019-04254).

## 3. Results

Data were collected from 336 case presentations and case discussions: 102 in neuro-oncology, 112 in soft-tissue sarcoma and 122 in hepatobiliary cancer. In the 101 cases with duplicate information from two observers, concordant themes were identified in 94/101 (93%) case presentations and discussions. 

Treatment recommendations were provided in 92% of the case discussions.

In the total sample set, information on physical status was reported in 48.2%, psychologic status in 8.9% and comorbidity in 48.5% (of which 44.3% reported comorbidity and 4.2% reported no significant comorbidity (Table 1)).

At least one nonmedical factor was referred to in 28.3% of the cases (Table 1). Among these, family relations were referred to in 7.7%, occupation in 5.7%, country of origin in 3.9% and abode in 3.6% of the cases. Patient preferences were reported in 4.2% of the cases. In 11.3% of the cases, valuations were given and were positive in 8.6% and negative in 2.7%, with examples provided in Table 2. 

**Figure 1:**
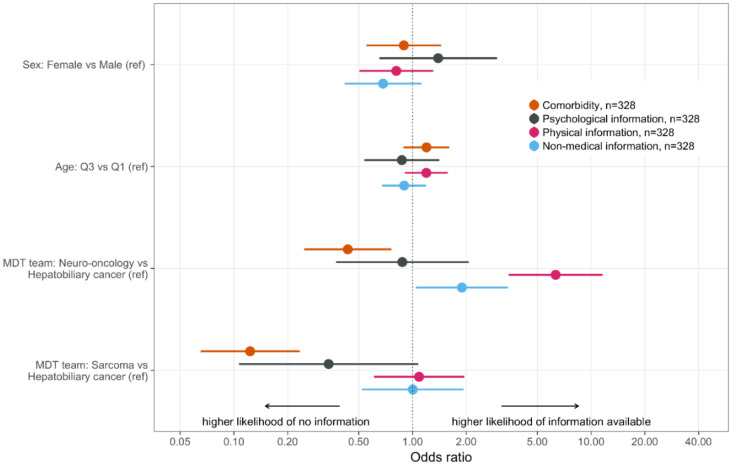
Odds ratios (ORs) for presenting comorbidity (orange), physical information (red), psychological information (black) and nonmedical information (blue) in relation to sex, age and MDT team. Whereas sex and age showed no influence on presentation of these factors, differences were discerned between the MDT teams. Physical and nonmedical information were more often provided in the neuro-oncology MDT, whereas presence of comorbidity was less frequently provided in the sarcoma MDT.

Information on medical factors, physical status, psychologic status and comorbidity did not correlate with age or sex (Figure 1, Table S1). Significant differences between the teams applied related to information on physical status, comorbidity and nonmedical factors (Figure 1, Table S1). Holding other parameters constant, information on physical status was more often reported at the MDT meetings for neuro-oncology compared to the hepatobiliary MDT meetings with an OR of 6.33 (3.46–11.6). Information on comorbidity was less often reported in the MDT meetings for soft-tissue sarcoma OR 0.12 (0.07–0.23) and neuro-oncology OR 0.43 (0.25–0.76) compared to the hepatobiliary MDT meeting. Information on psychologic factors did not show significantly differ between the teams, though we observed a trend for less frequent reporting on psychologic aspects in the MDT meetings for sarcoma compared to hepatobiliary cancer (Figure 1, Table S1). 

Reports of nonmedical information were more common in the MDT meeting for neuro-oncology, OR 1.89 (1.04–3.42), compared to the hepatobiliary MDT meeting. Analyses of specific types of nonmedical information were not performed due to restricted sample size. 

## 4. Discussion

A pertinent patient profile with complete and high-quality medical information and relevant nonmedical patient characteristics has been defined as a key component in robust decision-making and has been linked to a high likelihood for implementation of the recommendations provided [3,5,6,10,13]. Consequently, inadequate or lack of information on case history, comorbidities and patient´s preferences have been defined as major barriers to implementation of MDT recommendations [14,15,16]. We aimed to map to what extent patient-related medical and nonmedical characteristics are presented during MDT meetings in cancer care and can thereby be considered during collective decision-making. The diagnostic areas studied, i.e., neuro-oncology, hepatobiliary cancer and soft-tissue sarcoma, have broadly implemented MDT meetings to define the best possible treatment option, though the evidence for clinical benefit from MDT-based case management in these diagnostic areas is limited [17,18]. The MDTs studied typically discuss complex cases that require input from various diagnostic specialties, consideration of alternative and advanced treatment options within surgery, radiotherapy, medical oncology and palliative care, patient eligibility for clinical trials and decisions related to curative vs. palliative treatment intention. 

### 4.1. Medical Information

A comprehensive case presentation requires a structured and condensed provision of relevant medical information on the diagnostic path, including information on symptoms, biomarker measures, imaging results, pathology, tumor stage and definition of treatment-related considerations. Presentation of patient characteristics should efficiently capture the essence of risk factors, performance status, information on relevant comorbidities and available information on patient preferences [19,20,21]. Though many MDT teams have performed targeted quality improvement work related to, e.g., referral guidelines, meeting structure and case discussion format, there is a lack of guidelines on which data elements should be provided for a comprehensive MDT case discussion. Relevant information differs among teams, diagnostic areas and in relation to the clinical question, and to ensure efficient processes the data requested should be kept at a modest amount [13,21,22]. 

In our series, information on physical status, comorbidity and psychological status was provided in 48.2%, 48.5% and 8.9% of the cases respectively, which is comparable to previous observations [7,11]. The MDT for hepatobiliary cancer did significantly more often report on comorbidity, which may be explained by treatment discussions that often considered major surgery combined with chemotherapy and/or radiotherapy. Reports on comorbidity were, however, often ambiguous or vague and did generally not use comorbidity indices or scores. Information on e.g., chronic diseases such as hypertension or diabetes may be difficult to interpret without information on how well-regulated the disease is and whether severe side-effects or complications exist. Patient’s physical status was more often reported in the MDT for neuro-oncology compared to hepatobiliary cancer (Figure 1). This observation most likely reflects the profound effect that CNS tumors may have on general support needs and ability to manage daily life. Defined patient profiles support relevant decision-making. Between 10% and 30% of the treatment recommendations from MDTs have been found not to be implemented and deviations from MDT recommendations also risk significant delays in start of treatment [19,23]. Our observations thus suggest that structures for reporting on physical function, comorbidities, psychological factors and support needs are relevant to develop.

### 4.2. Nonmedical Characteristics

Patients’ nonmedical characteristics are frequently referred to in clinical practice, which has been documented e.g., related to judgements on pain medication [24,25,26]. We identified reporting of nonmedical factors such as family relations, occupation, country of origin and abode in 3.6–7.7% of the cases (Table 1). Previous studies, in e.g., breast cancer, gastrointestinal cancer, hematological and lymphatic diseases have demonstrated mentioning of psychological factors and social support structures at similar levels of 2–13% [7,11]. Certain nonmedical information may be relevant, e.g., occupation related to treatments that may risk impaired physical function and country of origin related to need for interpretation services, but could be avoided when irrelevant in the context of the medical situation and decision-making. As an example, occupation was in our series reported on in 19 cases, of which six referred to the patient being a health professional, which should not influence the clinical decision-making. 

### 4.3. Patient Preferences

Several studies have documented that patient preferences are rarely discussed in cancer-related MDT meetings, which also fits with our observations of patient preferences mentioned in 4.2% of the cases (Table 1) [4,5,7]. Limited consideration of patient perspectives has also been documented in individual consultations, e.g., in a study on treatment decisions related to preoperative radiotherapy for rectal cancer where patients’ values and treatment preferences were voiced in 22% of the cases [27]. Further, patients have limited information on MDT meetings and restricted opportunities to influence the decision-making process [28]. Patient perspectives have obvious relevance for successful implementation of the recommendations provided. However, it remains unclear at which stage of an MDT case discussion such preferences should be expressed, i.e., whether medical perspectives and clinical options should first be explored, followed by information on patients’ views or whether patient perspectives should be voiced prior to the case discussion on various treatment options [10]. Limited focus on patient factors and lack of involvement and representation at MDT meetings may be stressful for patients and may negatively influence satisfaction with the treatment recommendations provided. Hence, MDTs should consider strategies to ensure patient involvement and support informed MDT choices [29,30]. 

We found that patient preferences were randomly mentioned and referred to during MDT meetings, which may suggest a need for more knowledge and better structures for how and when to consider patient preferences in the MDT decision-making process. Robust MDT decisions provide an important basis for shared decision-making, which implies a shift in focus from the healthcare provider to the patient. Access to complete information, consideration of patient needs, recognition of barriers for implementation, and balancing risk and benefits support provision of treatment recommendations that are found appropriate and acceptable by the patient [31].

### 4.4. Patient-Related Valuations

The identification of valuation in 11.3% of the case discussions should in our opinion alert the MDT teams to the background and rationale for providing such statements as exemplified in Table 2. The identification of positive and negative valuations in 8.6% and 2.7% of the cases, respectively, is comparable to observations from France. Restivo et al. investigated how nonmedical patient-related information contributes to medical decision-making in MDTs and observed valuations related to the patients’ psychological presentation and relational perspectives [11]. Valuations typically do not provide information relevant to the case discussions and may indeed be provocative from the patient’s perspective. 

### 4.5. Weaknesses

The study was designed to map the frequency and types of mentioning of medical and nonmedical characteristics, but does not allow for conclusions on cause or effect hereof related to decision-making quality, implementations of the recommendations or patient outcomes. The study context was Swedish cancer care, which limits generalizability, though similar results have been reached in other countries [11]. Further, we cannot exclude that information on, e.g., comorbidity, patient characteristics and patient preferences were not available in clinical files, though they were not mentioned or alluded to in the MDT case discussions.

### 4.6. Strengths

Double observations with independent data collection were performed in one-third of the cases with 93% agreement, which supports consistent identification and reproducible classification of information categories. The risk of the Hawthorn effect was reduced by the MDT participants being informed that the aims of the observational assessment were MDT leadership and decision-making, without details that could have influenced case presentations and discussions. Further, the observer(s) did not interact in the meetings and typically participated from a position in the back of the room. The study contributes to the literature on the basis for decision-making in MDTs in cancer care.

## 5. Conclusions

MDT-based decision-making in cancer care requires access to and consideration of multiple and complex information. Various disciplines, professions and personalities should voice diagnostic and treatment-related perspectives and integrate patient-related factors and preferences. Though patient characteristics are key determinants of implementation of treatment recommendations from MDTs, we demonstrated that information on comorbidities is provided in about half of the case presentations and discussions while patient characteristics and patient preferences are mentioned in <10% of the cases. Definition of data elements and development of consensus reporting standards could be relevant to further develop efficient MDT decision-making. Establishment of checklists could support data collection and reporting, and contribute to relevant, complete and efficient case presentations and discussions [32]. Our observations suggest that initiatives to further improve information structures at MDT meetings should be considered to ensure high-quality decision-making and treatment recommendations with a high likelihood for implementation.

## Figures and Tables

**Table 1:** Summary of information on medical and nonmedical information provided.

Patient Characteristics and Factors Registered		Neuro-Oncology	Hepatobiliary Cancer	Sarcoma	Total
Sex					
Female		48 (47.1%)	58 (47.9%)	45 (40.2%)	151 (45.1%)
Male		54 (52.9%)	63 (52.1%)	67 (59.8%)	184 (54.9%)
Missing		0	1	0	1
Age Median (IQR)		65 (54–72)	71 (61–64)	56 (38–69)	66 (54–73)
Min–max		22–86	31–85	0–95	0–95
Missing		0	1	6	7
Medical information					
Comorbidity		52 (50.9%)	87 (71.4%)	24 (21.5%)	163 (48.5%)
No significant comorbidity present		8 (7.8%)	3 (2.5%)	3 (2.7%)	14 (4.2%)
Comorbidity present		44 (43.1%)	84 (68.9%)	21 (18.8%)	149 (44.3%)
Physical status		79 (77.5%)	45 (36.9%)	38 (33.9%)	162 (48.2%)
Psychological status		11 (10.8%)	14 (11.5%)	5 (4.5%)	30 (8.9%)
Nonmedical information					
Any nonmedical information		38 (37.3%)	28 (23 %)	29 (25.9%)	95 (28.3%)
Abode		9 (8.8%)	2 (1.6%)	1 (0.9%)	12 (3.6%)
Occupation		7 (6.9%)	6 (4.9%)	6 (5.4%)	19 (5.7%)
Country of origin		6 (5.9%)	4 (3.3%)	3 (2.7%)	13 (3.9%)
Patient preferences		8 (7.8%)	3 (2.5%)	3 (2.7%)	14 (4.2%)
Family relations		14 (13.7%)	4 (3.3%)	8 (7.1%)	26 (7.7%)
Valuation	Positive	7 (6.9%)	14 (11.5%)	8 (7.1%)	29 (8.6%)
	Negative	4 (3.9%)	1 (0.8%)	4 (3.6%)	9 (2.7%)

**Table 2:** Examples of positive and negative valuations.

Positive Valuations	Negative Valuations
Very nice person	Not easy to deal with
Very young and active woman	Much drama around the patient
Very nice gentleman	Not the healthiest person we met
“Bloody” active	Asked me and the rest of the world
Has a sensible wife	Challenging patient
Patient related to (famous person)	The patient is “semi-functional”
Feeling great, works 150%, good-looking	

## Data Availability

Raw data are provided by the authors upon request.

## Supplementary Materials

The following are available online at https://www.mdpi.com/1718-7729/28/1/98/s1, Table S1: Data to support Figure 1, correlations and odds ratios for information in relation to patient characteristics and MDT team.
