# Supplementary material for: Medical and Nonmedical Information during Multidisciplinary Team Meetings in Cancer Care
Source: Curr Oncol. 2021 Feb 23;28(1):1008–16. doi: 10.3390/curroncol28010098 (PMC7985788; doi:10.3390/curroncol28010098)
Supplement: Supplementary file 1 [file curroncol-28-00098-s001.pdf]

Supplementary table 1. Data to support figure 1; correlations and odds ratios for information in relation to patient characteristics and MDT team.

		Odds Ratio (95% CI)
**Physical information**		
	Age: Q3 vs Q1 (ref)	1.19 (0.91-1.58)
	Sex: Female vs Male (ref)	0.81 (0.5-1.3)
	Team: Neuro-oncology vs Hepatobiliary cancer (ref)	6.33 (3.46-11.6)
	Sarcoma vs Hepatobiliary cancer (ref)	1.09 (0.61-1.95)
**Comorbidity**		
	Age: Q3 vs Q1 (ref)	1.2 (0.89-1.61)
	Sex: Female vs Male (ref)	0.89 (0.55-1.45)
	Team: Neuro-oncology vs Hepatobiliary cancer (ref)	0.43 (0.25-0.76)
	Sarcoma vs Hepatobiliary cancer (ref)	0.12 (0.07-0.23)
**Psychological information**		
	Age: Q3 vs Q1 (ref)	0.87 (0.54-1.41)
	Sex: Female vs Male (ref)	1.39 (0.65-2.97)
	Team: Neuro-oncology vs Hepatobiliary cancer (ref)	0.88 (0.37-2.06)
	Sarcoma vs Hepatobiliary cancer (ref)	0.34 (0.11-1.08)
**Non-medical information**		
	Age: Q3 vs Q1 (ref)	0.9 (0.68-1.19)
	Sex: Female vs Male (ref)	0.68 (0.42-1.12)
	Team: Neuro-oncology vs Hepatobiliary cancer (ref)	1.89 (1.04-3.42)
	Sarcoma vs Hepatobiliary cancer (ref)	1 (0.52-1.93)
